# Basiliximab in the Prophylaxis of aGVHD for Unrelated Donor Hematopoietic Stem Cell Transplantation in Patients With Thalassemia Major: A Prospective, Multicenter, Open‐Label, Randomized Controlled Study

**DOI:** 10.1002/ajh.70169

**Published:** 2025-12-19

**Authors:** Zhenbin Wei, Rongrong Liu, Lingling Shi, Qiaochuan Li, Lianjin Liu, Baoshi Zheng, Chunjie Qin, Hong Chen, Guyun Wang, Meiqing Wu, Gaohui Yang, Ruolin Li, Zhaoping Gan, Qi Zhou, Jing Fan, Xuemei Zhou, Yinghua Chen, Zhiyu Zeng, Zhongming Zhang, Yongrong Lai

**Affiliations:** ^1^ Department of Hematology The First Affiliated Hospital of Guangxi Medical University Nanning Guangxi China; ^2^ Guangxi Key Laboratory of Thalassemia Research Nanning China; ^3^ NHC Key Laboratory of Thalassemia Medicine Nanning China; ^4^ Department of Hematology Liuzhou Worker's Hospital Liuzhou Guangxi China; ^5^ Department of Hematology Hainan Provincial People's Hospital Haikou Hainan China; ^6^ Department of Health Research Methods, Evidence, and Impact McMaster University Hamilton Ontario Canada

To the Editor,

We present the first prospective, multicenter, randomized controlled trial (RCT) evaluating Basiliximab prophylaxis for acute graft‐versus‐host disease (GVHD) in thalassemia major (TM) patients undergoing Matched unrelated donor hematopoietic stem‐cell transplantation (MUD‐HSCT). This study was registered at ClinicalTrials.gov [#NCT02342145]. This study addresses a critical unmet need in optimizing GVHD prevention strategies in this high‐risk population.

Allogeneic hematopoietic stem‐cell transplantation (allo‐HSCT) remains the only curative therapy currently available for thalassemia major (TM). While HLA‐matched sibling‐donor (MSD) transplantation yields > 90% overall survival (OS) and > 80% thalassemia‐free survival (TFS), only 25%–30% of patients have available MSD [1, 2]. Matched unrelated donor (MUD) transplantation thus represents a common alternative, but graft‐versus‐host disease, graft rejection and transplant‐related mortality (TRM) remain obstacles.

Basiliximab, an IL‐2 receptor (CD25) antagonist, has demonstrated efficacy in solid organ transplant rejection prophylaxis and in steroid‐refractory aGVHD. Our trial rigorously compares standard GVHD prophylaxis with or without Basiliximab to clarify its prophylactic role in unrelated donor HSCT.

Three Chinese transplant centers enrolled patients (April 2015–September 2021) with transfusion‐dependent TM, adequate organ function and no active infection or hepatitis. Key exclusions were HIV positivity, cytomegalovirus (CMV) or Epstein–Barr virus (EBV) viremia > 200 copies/ml, or transaminases > 4 × ULN. Written parental consent was obtained.

After eligibility confirmation, patients were sequentially numbered per center; alternate numbers were assigned to Basiliximab or control arms (open‐label). All received identical myelo‐ablative conditioning: fludarabine 50 mg/m^2^ days −12 to −10, busulfan 1 mg/kg q6 h days −9 to −6, cyclophosphamide 50 mg/kg days −5 to −2 and anti‐thymocyte (ATG, Thymoglobulin) 2.5 mg/kg days −4 to −1. Hydroxyurea 30 mg/kg had been given for 2 months pre‐conditioning. Peripheral‐blood stem cells were infused from 10/10 or 9/10 HLA matching MUDs. GVHD prophylaxis in both arms consisted of tacrolimus (FK506 0.03 mg/kg/day, target 5–15 ng/mL) from day −3, methotrexate (15 mg/m^2^ day +1, 10 mg/m^2^ days +3, +6, +11) and mycophenolate mofetil (MMF 250 mg/day) for 90 days. Basiliximab‐arm patients additionally received 10 mg (< 35 kg) or 20 mg (≥ 35 kg) intravenously on days 0 and +4. Follow‐up included weekly evaluations to day +100, fortnightly to month 6, then monthly. CMV/EBV PCR, blood counts, chimerism and GVHD assessments were performed centrally.

The primary endpoint was cumulative incidence of grade II‐IV aGVHD during day 100, determined by the transplant specialist team in accordance with the Modified Glucksberg Grading. Secondary endpoints were grade III‐IV aGVHD, chronic GVHD (cGVHD), neutrophil and platelet engraftment, CMV/EBV reactivation, bacterial, fungal infections, TRM, OS, and thalassemia‐free survival (TFS). Exploratory analyses examined patient age, donor age, sex mismatch, and HLA disparity.

Sample size was calculated to detect a reduction in grade II‐IV aGVHD from 35.4% (historical control) to 22.5% (two‐sided, α = 0.05, power 80%). Allowing 6.3% dropout, 205 patients (≈102 per arm) were required. Comparisons used χ^2^ or Mann–Whitney U tests for categorical/continuous variables. Competing‐risk regression (Fine‐Gray model) assessed aGVHD and cGVHD incidences with graft failure, relapse, and death as competing events. Survival analyses employed Kaplan–Meier curves and Cox models. For binary variables, odds ratio (OR) and 95% CI, logistic regression was used.

205 patients were randomized (Basiliximab *n* = 102; control *n* = 103); all completed transplantation and were analyzed (Figure S1). Median age 7 years (IQR 4–9). Baseline characteristics were balanced except for a slight male excess in controls (Table 1). The follow‐up data cut‐off date is 20 August 2022 (median follow‐up time was 32.3 months). Median time for neutrophils engraftment was 11 days in both arms (*p* = 0.40); median time for platelets engraftment occurred at 12 days in each arm (*p* = 0.57) (Table S1).

**TABLE 1:** Patient characteristics at baseline.

Variable	Total	Basiliximab group	Control group	
	*N* = 205	*N* = 102	*N* = 103	*p*
Sex				
Male	121 (59.0%)	53 (52.0%)	68 (66.0%)	0.04
Female	84 (41.0%)	49 (48.0%)	35 (34.0%)	
Age, years				
Median (IQR)	7 (4–9)	7 (4–9)	7 (5–10)	0.10
Age < 7	93 (45.3%)	49 (48.0%)	44 (42.7%)	0.44
Age ≥ 7	112 (54.6%)	53 (52.0%)	59 (57.3%)	
Splenectomy	19 (9.3%)	8 (7.8%)	11 (10.7%)	0.48
Serum ferritin, ng/mL				
Median (IQR)	3642 (2464.1–5216.6)	3686.0 (2682.1–5361.5)	3601.24 (2356.5–4867.0)	0.46
< 2500	51 (25%)	22 (22%)	29 (28%)	0.28
≥ 2500	154 (75%)	80 (78%)	74 (72%)	
Donor age, years				
Age < 40	35 (17.1%)	20 (19.6%)	15 (14.6%)	0.34
Age ≥ 40	170 (82.9%)	82 (80.4%)	88 (85.4%)	
Female donor for male				
Yes	33 (16.1%)	17 (16.67%)	16 (15.53%)	0.83
No	172 (83.9%)	85 (83.33%)	87 (84.47%)	
HLA				
HLA 10/10 matched	180 (87.8%)	86 (84.3%)	94 (91.3%)	0.13
HLA 9/10 matched	25 (12.2%)	16 (15.7%)	9 (8.7%)	
Total MNCs, × 10^8^/kg, median (IQR)	15.1 (12.0–18.4)	15.2 (12.4–18.5)	14.8 (11.5–18.3)	0.52
Toal CD34^+^cells, × 10^6^/kg, median (IQR)	8.44 (6.3–12.0)	8.4 (6.1–12.2)	8.6 (6.7–11.8)	0.74

Abbreviations: HLA, human leukocyte antigen; MNC, mononuclear cell.

The cumulative incidence of grade II–IV aGVHD by day 100 was 25.5% in the Basiliximab arm versus 31.1% in the control arm (HR 1.21; 95% CI 0.73–2.03; *p* = 0.46) (Table S1, Figure 1B), showing no statistically significant difference. Grade III–IV aGVHD was numerically lower in the Basiliximab group (8.8% vs. 14.5%; *p* = 0.21) (Table S1, Figure 1C). Univariate analysis identified no significant associations between patient age, donor age, donor‐recipient sex mismatch, or HLA matching and the incidence of acute GVHD (Table S2). A post hoc subgroup analysis was done based on HLA matching status (9/10 vs. 10/10). Results showed no statistically significant differences in aGVHD and cGVHD incidence rates between the 9/10 HLA‐matched group and 10/10 HLA‐matched group (Figure S2A–D).

**FIGURE 1:**
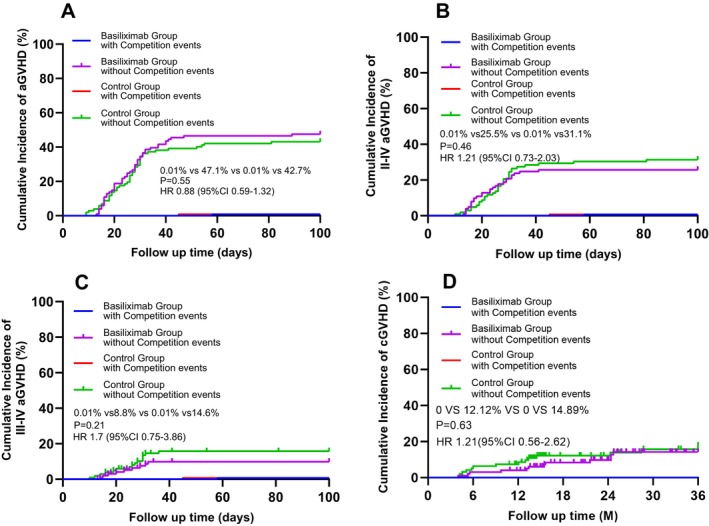
Competing risk model for cumulative incidence of aGVHD (A), II–IV aGVHD (B), III–IV aGVHD (C), cGVHD (D).

Three‐year OS was 97.06% with Basiliximab versus 92.23% in controls (HR 0.37; *p* = 0.12) (Table S1, Figure 2A,D). TRM was significantly reduced with Basiliximab (1.96% vs. 7.77%; HR 0.25; *p* = 0.05) (Table S1, Figure 2D,F). In the Basiliximab group, three cases resulted in death, while eight deaths were recorded in the control group (Table S3). All survivors remained transfusion‐independent, highlighting encouraging clinical trends despite a negative primary endpoint.

**FIGURE 2:**
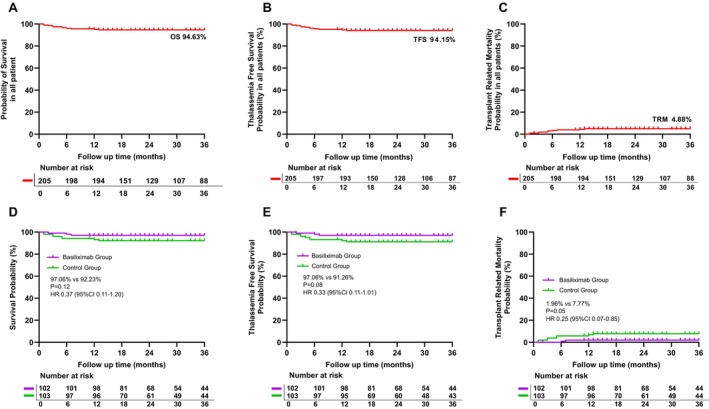
Kaplan–Meier curves for overall survival, thalassemia‐free survival, and transplant‐related mortality by all patients and two groups.

Overall infection incidence was similar (Basiliximab 73.53% vs. control 65.05%; OR 0.67; 0.37–1.22; *p* = 0.19) (Table S1). CMV reactivation (34.31% vs. 33.98%), EBV reactivation (9.80% vs. 8.74%), fungal infection (10.78% vs. 5.83%), septicemia (11.76% vs. 9.71%), and pneumonia (18.63% vs. 13.59%) did not differ (Table S1). Bacterial infections were more frequent in the Basiliximab group (56.86% vs. 41.75%; OR 0.54; 0.31–0.95; *p* = 0.03) (Table S1). Rates of veno‐occlusive disease, hemorrhagic cystitis, and autoimmune cytopenias were similar between the two groups (Table S1).

This first RCT indicates that administering Basiliximab on days 0 and + 4, in addition to standard FK506/MTX/MMF prophylaxis, does not significantly decrease the occurrence of grade II‐IV or III‐IV aGVHD following MUD‐HSCT for thalassemia major. Nevertheless, the antibody was safe, did not delay engraftment, and showed encouraging trends for superior TRM, OS, and TFS.

Several factors may explain this negative finding. First, the dosing regimen of Basiliximab (20 mg on days 0 and + 4) was adapted from a protocol originally developed for renal transplant rejection prophylaxis [3]. Retrospective studies have reported potential benefits of this regimen for the prophylaxis of GVHD in patients undergoing MUDs or haploidentical allo‐HSCT [4, 5]. However, these studies lacked prospective randomization, resulting in insufficient evidence for dosing effectiveness in allo‐HSCT. A phase II trial utilizing a higher dose (40 mg on day +9) suggested that alternative dosing strategies might be necessary to achieve optimal effectiveness [6]. Thus, the current regimen may have failed to maintain adequate drug exposure during the critical window for aGVHD prevention. Second, Basiliximab targets the alpha‐chain (CD25) of the IL‐2 receptor on activated T cells, thereby inhibiting IL‐2‐mediated proliferation of donor‐derived activated T lymphocytes, which are central to GVHD pathophysiology [7, 8, 9]. However, Basiliximab has no effect on resting T cells, which do not express CD25 [8]. Pharmacokinetic data indicate that Basiliximab has a half‐life of approximately 6.5 ± 2.1 days, with therapeutic serum concentrations (> 0.2 μg/mL) sustained for about 26 ± 8 days following a 40 mg dose [10]. Given that the median onset of aGVHD in our cohort was day +26 (range, +9 to +117), the current dosing schedule likely resulted in subtherapeutic drug levels during the peak risk period. Future studies should consider adjusted dosing or timing to prolong therapeutic exposure.

Despite these limitations, we observed encouraging trends in secondary outcomes. The 3‐year OS and TFS for the entire cohort were 94.63% and 94.15%, respectively, comparable to outcomes reported for MSD transplants. Although the primary endpoint was not met, Basiliximab was associated with improved OS (97.06% vs. 92.23%) and TFS (97.06% vs. 91.26%), and a more outstanding improvement in TRM (1.96% vs. 7.77%; HR 0.25; 0.07–0.85; *p* = 0.05) (although it is at the threshold, there is a favorable trend). These findings suggest a potential protective effect against severe transplant‐related complications, possibly through attenuation of high‐grade GVHD or other immune‐mediated toxicities.

Nonetheless, the incidence of grade II–IV (28.29%) and grade III–IV (11.71%) aGVHD remained high compared to our prior MSD‐HSCT cohort (15.3% and 6.0%, respectively) [2], underscoring the persistent challenge of GVHD in the MUD setting. These rates are consistent with published data from other centers reporting aGVHD incidences of 20%–60% in TM patients undergoing MUD‐HSCT [1, 2, 11, 12, 13, 14, 15]. Thus, while Basiliximab did not meet the intended primary endpoint, these findings underscore the potential clinical benefit of modifying the regimen to optimize its immunosuppressive effect, which merits further investigation in future prospective trials.

Importantly, Basiliximab was well tolerated, with no significant infusion‐related adverse events. There were no differences between groups in time to neutrophil or platelet engraftment or overall toxicity. Despite significant differences in bacterial infection incidence, the majority were promptly and effectively controlled. Additionally, the incidence of other infectious complications, including CMV and EBV reactivations, fungal infections, septicemia, or pneumonia, is concerning. These data support the safety of Basiliximab in this setting, although its immunosuppressive potency may require enhancement through dose or schedule modification.

In conclusion, while Basiliximab did not significantly lower aGVHD incidence, it was safe, well‐tolerated, and associated with improved TRM and survival outcomes. These findings support further investigation into optimized dosing and timing strategies to maximize its prophylactic potential in MUD‐HSCT for TM.

## Author Contributions

Y.L., Q.L., and Z.Z. designed the study, accessed and verified the data, analyzed the results. Z.W., R.L., and L.S. treated the patients, performed the statistical analysis, and wrote the manuscript. L.L., C.Q., H.C., G.W., M.W., G.Y., Z.G., J.F., X.Z., and Y.C. advised on the protocol, treated the patients, analyzed the results, and reviewed the manuscript. All authors had access to the statistical reports, and all had final responsibility for the decision to submit for publication.

## Funding

The research was supported by the Young Leader Talent Training Program of Guangxi Medical University (No. 202302), the Advanced Innovation Teams and Xinghu Scholars Program of Guangxi Medical University, the Joint Project on Regional High‐Incidence Diseases Research of Guangxi Natural Science Foundation (Grant No. 2023GXNSFAA026293), the NHC Key Laboratory of Thalassemia Medicine, and the Guangxi Key Laboratory of Thalassemia Research.

## Ethics Statement

All procedures were carried out by the relevant guidelines. This study was approved by the ethics committee of the First Affiliated Hospital of Guangxi Medical University [approval number: gxmuh‐2014‐14]. Written informed consent to participate in this study was obtained from the patients. Adults (≥ 18) finished the informed consent by themselves. For minors (< 18), informed consent was provided by the guardians on behalf of the minors involved in this study. Consent for publication: All patients and guardians agreed to the publication of this research.

## Conflicts of Interest

The authors declare no conflicts of interest.

## Supporting information


**Data S1:** Supporting Information.

## Data Availability

Original data may be made available for academic research upon reasonable request to the corresponding author.
