# Supplementary material for: Basiliximab in the Prophylaxis of aGVHD for Unrelated Donor Hematopoietic Stem Cell Transplantation in Patients With Thalassemia Major: A Prospective, Multicenter, Open‐Label, Randomized Controlled Study
Source: Am J Hematol. 2025 Dec 19;101(3):628–32. doi: 10.1002/ajh.70169 (PMC12868989; doi:10.1002/ajh.70169)
Supplement: Supplementary file 1 — Data S1: Supporting Information. [file AJH-101-628-s001.pdf]

SUPPORTING INFORMATION FOR

Basiliximab in the Prophylaxis of aGVHD for Unrelated Donor Hematopoietic Stem Cell Transplantation in Patients with Thalassemia Major: A Prospective, Multicenter, Open-Label, Randomized controlled Study

Table S1. Transplantation Outcomes

Variable	Total patients N=205	Basiliximab group N=102	Control group N=103	*P*
Neutrophil recovery, days				
Median (range)	11 (9-22)	11 (9-22)	11 (9-20)	
Difference, median (95% CI)		--	0 (0.0-1.0)	0.40
Platelet recovery, days				
Median (range)	12 (7-66)	12 (8-66)	12 (7-34)	
Difference, median (95% CI)		--	0 (-1.0-1.0)	0.57
Graft Failure				
n (%)	1 (0.48)	0 (0)	1 (0.97)	--
aGVHD at 100 days				
n (%)	92 (44.88)	48 (47.06)	44 (42.72)	
HR (95% CI)		--	0.88 (0.59-1.32)	0.55
aGVHD grade II-IV at 100 days				
n (%)	58 (28.29)	26 (25.49)	32 (31.07)	
HR (95% CI)		--	1.21 (0.56-2.03)	0.46
aGVHD grade III-IV at 100 days				
n (%)	24 (11.71)	9 (8.82)	15 (14.56)	
HR (95% CI)		--	1.7 (0.75-3.86)	0.21
cGVHD at 3 years				
n (%)	26(12.68)	12(12.12)	14 (14.89)	
HR (95% CI)		--	1.21(0.56-2.62)	0.63
Moderate-severe cGvHD c, n				
n (%)	17 (8.30)	8 (7.84)	9 (8.73)	0.92
HR (95% CI)		--	1.05 (0.42-2.64)	
OS at 3 years				
n (%)	194 (94.63)	99 (97.06)	95 (92.23)	
HR (95% CI)		--	0.37 (0.11-1.20)	0.12
TFS at 3 years				
n (%)	193 (94.15)	99 (97.06)	94 (91.26)	
HR (95% CI)		--	0.33 (0.11-1.01)	0.08
TRM at 3 years				
n (%)	10 (4.88)	2 (1.96)	8 (7.77)	
HR (95% CI)		--	0.25 (0.07-0.85)	0.05
Graft versus host disease and relapse-free survival, n				
n (%)	168 (82.0)	88 (86.3)	80 (77.7)	0.08
HR (95% CI)			1.79 (0.92-3.49)	
Infection				
n (%)	142 (69.27)	75 (73.53)	67 (65.05)	
OR (95% CI)		--	0.67 (0.37-1.22)	0.19
CMV Reactivation				
n (%)	70 (34.15)	35 (34.31)	35 (33.98)	
OR (95% CI)		--	0.99 (0.55-1.76)	0.96
EBV Reactivation				
n (%)	19 (9.27)	10 (9.80)	9 (8.74)	

OR (95% CI)		--	0.88 (0.34-2.27)	0.79
**Bacterial Infection**				
n (%)	101 (49.27)	58 (56.86)	43 (41.75)	
OR (95% CI)		--	0.54 (0.31-0.95)	0.03
**Fungal Infection**				
n (%)	17 (8.29)	11 (10.78)	6 (5.83)	
OR (95% CI)		--	0.51 (0.18-1.44)	0.20
**Septicemia**				
n (%)	22 (10.73)	12 (11.76)	10 (9.71)	
OR (95% CI)		--	0.81 (0.33-1.96)	0.63
**Pneumonia**				
n (%)	33 (16.10)	19 (18.63)	14 (13.59)	
OR (95% CI)		--	0.69 (0.32-1.46)	0.33
**VOD/SOS**				
n (%)	11 (5.36)	5 (4.90)	6 (5.83)	
OR (95% CI)		--	1.20 (0.35-4.06)	0.77
**HC**				
n (%)	57 (27.80)	30 (29.41)	27 (26.21)	
OR (95% CI)		--	0.85 (0.46-1.57)	0.61
**Post-transplant autoimmune anemia**				
n (%)	4 (1.95)	1 (0.98)	3 (2.91)	
OR (95% CI)		--	3.03 (0.31-29.62)	0.62

Abbreviations: aGvHD, acute graft versus host disease; cGvHD, chronic graft versus host disease; OS, overall survival; HR, hazard ratio; CI, confidence interval; TFS, thalassemia-free survival; TRM, transplant related mortality; OR, odds ratio; CMV, cytomegalovirus; EBV, Epstein-Barr virus; VOD/SOS, hepatic veno-occlusive disease/ sinusoidal obstruction syndrome; HC, hemorrhagic cystitis.

Table S2.Univariate analysis of aGVHD

	All Patients			Control Group			Basiliximab Group		
Variable	Events/evaluable	HR (95% CI)	*P*	Events/evaluable	HR (95% CI)	*P*	Events/evaluable	HR (95% CI)	*P*
**aGVHD grade II-IV at 100 days**									
Age at transplantation		0.95 (0.56-1.59)	0.84		0.99 (0.49-2.01)	0.98		0.93 (0.43-1.99)	0.85
< 7 years	33/112			19/59			14/53		
≥ 7 years	25/93			13/44			12/49		
Age of donor		1.52 (0.82-2.82)	0.18		1.1 (0.43-2.81)	0.84		2.05 (0.90-4.65)	0.09
<40 years	45/170			27/88			18/82		
≥ 40 years	13/35			5/15			8/20		
Female for male		0.6 (0.25-1.46)	0.26		0.93 (0.27-2.56)	0.75		0.39 (0.09-1.72)	0.22
Yes	6/33			4/16			2/17		
No	52/172			28/87			24/85		
HLA typing		0.83 (0.39-1.75)	0.62		0.90 (0.29-2.89)	0.85		0.74 (0.28-1.94)	0.54
10/10 matched	50/180			29/94			21/86		
9/10 matched	8/25			3/9			5/16		
**aGVHD grade III-IV at 100 days**									
Age at transplantation		0.88 (0.39-1.97)	0.75		.0.69 (0.23-2.03)	0.50		1.38 (0.37-5.05)	0.63
< 7 years	14/112			10/59			4/53		
≥ 7 years	10/93			5/44			5/49		
Age of donor		0.99 (0.34-2.92)	0.99		0.89 (0.19-4.10)	0.87		0.62 (0.08-4.95)	0.65
<40 years	20/170			13/88			7/82		
≥ 40 years	4/35			2/15			2/20		
Female for male		0.76 (0.22-2.62)	0.66		0.90 (0.21-3.92)	0.88		1.22 (0.25-5.98)	0.80
Yes	3/33			2/16			1/17		
No	21/172			13/87			8/85		
HLA typing		0.48 (0.18-1.31)	0.15		0.58 (0.13-2.57)	0.47		0.33 (0.08-1.33)	0.12
10/10 matched	19/180			13/94			6/86		
9/10 matched	5/25			2/9			3/16		

Abbreviations: aGvHD, acute graft versus host disease; HR, hazard ratio; CI, confidence interval; HLA, human leukocyte antigen.

Table S3. Detailed cause of death

HLA type	Group	Sex	Time of death (post-transplantation)	Cause of death	
				Direct cause	Indirect cause
9/10	Basiliximab	Female	6 months	pulmonary hemorrhage	grade IV aGVHD, severe pneumonia
9/10	Basiliximab	Female	7 months	cerebral hemorrhage	decreased implantation, ineffective platelet transfusion
10/10	Basiliximab	Male	4 months	accidental fall to death	accidental fall to death
9/10	control	Male	45 days	respiratory failure	decreased implantation, severe pneumonia
9/10	control	Male	3 months	central infection	grade IV aGVHD, infection
10/10	control	Male	5 months	respiratory failure	severe pneumonia
10/10	control	Male	25 days	multiple organ dysfunction syndrome	severe sepsis
10/10	control	Male	13 months	infection	autoimmune hemolytic anemia, infection
10/10	control	Male	12 months	respiratory failure	cGVHD, bronchitis obliterans
10/10	control	Female	6 months	septic shock	grade IV aGVHD, severe sepsis
10/10	control	Female	3 months	respiratory failure	grade IV aGVHD, severe pneumonia

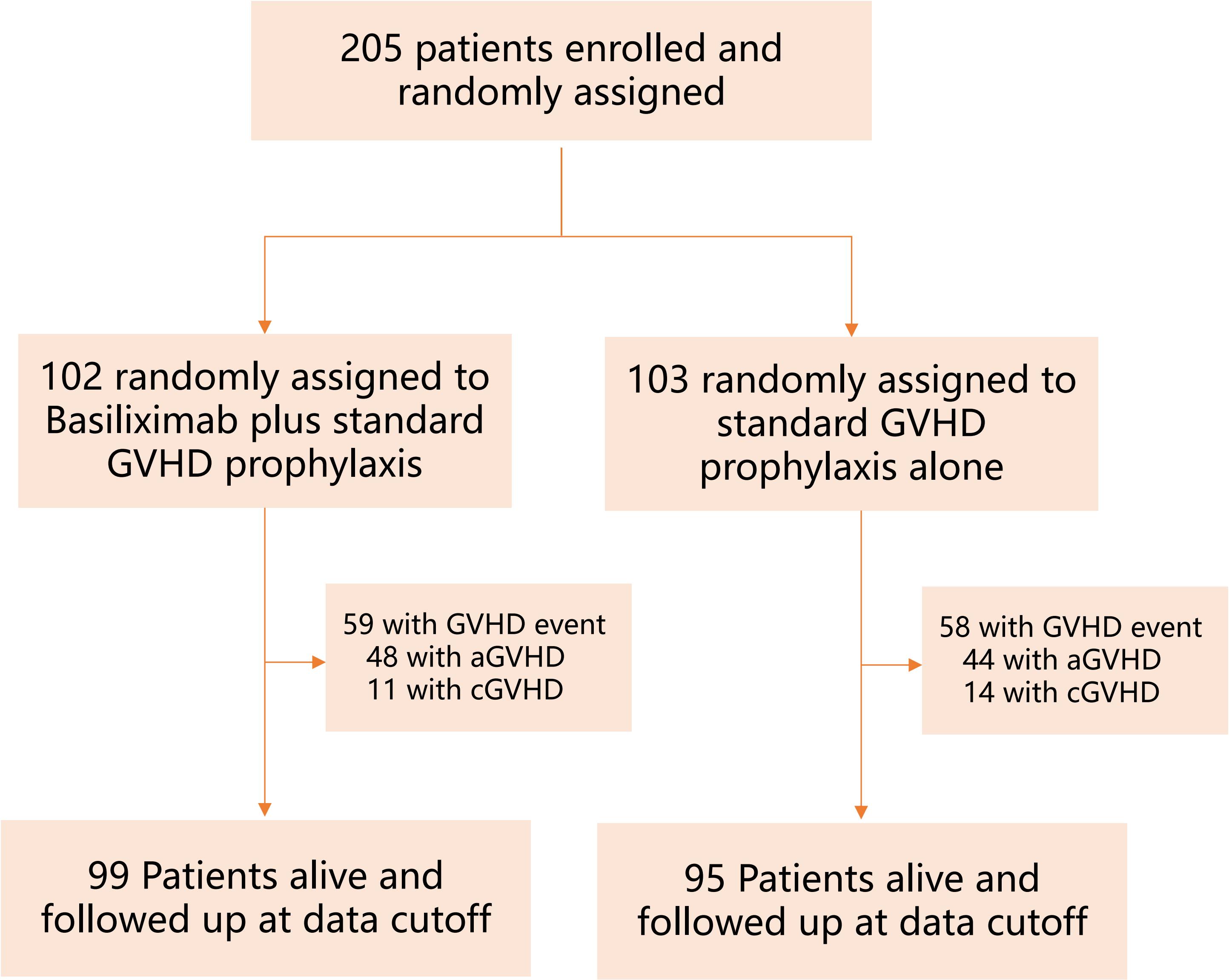

Figure S1.Trialprofile

No patients deviated from protocol, and no patients were lost to follow-up.

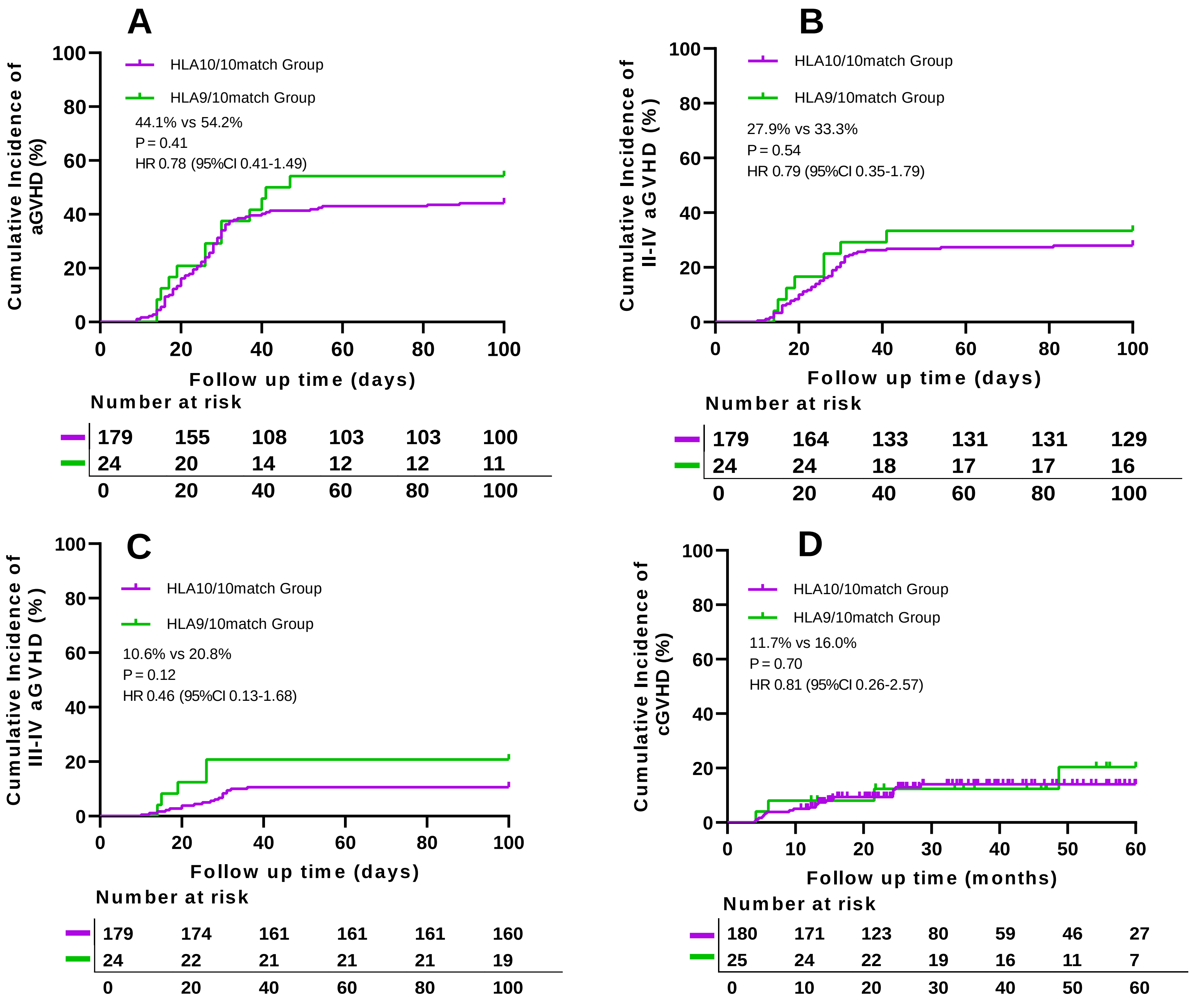

Figure S2. Post-hoc subgroup analysis ased on HLA-match for aGVHD (A), II-IV aGVHD (B), III-IV aGVHD (C), cGVHD (D)
